# A Case of Headache Treated by Online Telemedicine in Collaboration With a Midwifery Home

**DOI:** 10.7759/cureus.61203

**Published:** 2024-05-27

**Authors:** Masahito Katsuki, Mayumi S Kaido, Daiki Sato

**Affiliations:** 1 Physical Education and Health Center, Nagaoka University of Technology, Nagaoka, JPN; 2 Department of Neurosurgery, Tsubame-Sanjo Sugoro Neurospine Clinic, Sanjo, JPN; 3 Midwifery, NekoNeko Midwifery Home, Sado, JPN

**Keywords:** migraine, tension-type headache, midwife, operational efficiency, online nurse, visiting nurse, rural area, remote medical care, online telemedicine, headache

## Abstract

Midwifery centers are places where midwives not only provide antenatal checkups and delivery care but also offer a wide range of health guidance to pregnant women, postpartum mothers, newborns, and older women. In recent years, midwives have also provided onsite and online health guidance. However, diagnosis and prescribing medication are impossible in midwifery centers because no doctor is present. If the midwife determines that the patient should consult doctors, the patient may have to go to a hospital and see doctors in person, which can be burdensome. Online telemedicine facilitates midwife-doctor collaboration and may solve this problem.

We report a case of headache management by telemedicine that minimized the patient's travel burden by collaborating with a midwifery center that provides onsite, visiting, and online health guidance for patients who have difficulty visiting a hospital due to postpartum period, childcare, and breastfeeding. A 29-year-old woman and her husband were raising an infant in Sado City (a remote island across the sea), Niigata Prefecture. She developed acute back pain and was bedridden for several days due to immobility. She consulted a midwife because of stress and anxiety caused by childcare and acute back pain, as well as newly occurring headaches. The midwife visited her and provided on-site health guidance. The midwife decided that a doctor's diagnosis and treatment with painkillers were desirable for the headache and back pain, so she contacted a doctor based on the patient's request. The doctor provided online telemedicine across the sea, diagnosed her headache as a tension-type headache, and prescribed acetaminophen 500 mg as an abortive prescription. The prescription was faxed to a pharmacy on the island, and the original was sent by post. The midwife picked up the medication and delivered it to the patient. After taking the medication, the patient's back pain and headache went into remission. Collaboration between midwifery centers that provide onsite, visiting, and online health guidance and medical institutions that offer online telemedicine can potentially improve accessibility to medical care. It differs from conventional online telemedicine in the midwife’s coordination practice by monitoring the patient's condition and requesting the physician based on the patient's request.

## Introduction

Since March 2020, the coronavirus disease 2019 (COVID-19) pandemic has heightened the demand for telemedicine to reduce the necessity for in-person consultations [1,2]. Until May 8, 2023, COVID-19 was classified as a category 2 infectious disease in Japan. In addition to the need for public health centers to conduct full surveillance, numerous operational and medical resources, such as hospitalization recommendations and work restriction measures, were also required. Because there were not enough hospital beds, COVID-19 patients with mild symptoms were also given official instructions to stay home or receive hotel care. The large influx of outpatients with fever and COVID-19 patients strained the normal operations of medical facilities, and the hospitalization of COVID-19 patients also caused a shortage of beds. Hospitals were also physically restricted by separating fever patients from regular patients in the flow of patients from the entrance to waiting rooms and examination rooms. Under these circumstances, the ban on online medical services was officially lifted. Japan officially launched online telemedicine for most diseases in April 2022 [3]. Patients with fever were diagnosed and prescribed medicine by a doctor using an over-the-counter test kit without going to the hospital. Patients with lifestyle-related diseases and other regular hospital visits could also avoid unnecessary contact with other patients through online medical care. 

Although online telemedicine was initially initiated through this process, it is now being used in specialty outpatient clinics such as headache outpatient clinics [4,5]. The safety and effectiveness of virtual consultations for primary headaches are comparable to those of in-person consultations [6]. There is increasing anticipation that online telemedicine will also be used more with video-based consultations, appropriately combined with traditional face-to-face treatment.

Midwifery centers not only provide antenatal checkups and delivery care, but they also offer a variety of health guidance for pregnant women, postpartum mothers, newborns, and older women. Midwives have recently begun offering health guidance on-site (visiting patient’s home) and online [7]. However, because no doctors are present at these centers, the midwife cannot diagnose or prescribe medication. If a midwife determines that a patient needs to see a doctor, the patient often has to go to a hospital, which can be inconvenient. In other words, midwifery centers cannot diagnose or prescribe medication, necessitating hospital referrals, which can be cumbersome for patients. Online telemedicine can help address this issue by facilitating collaboration between midwives and doctors. That is, if a patient sought health guidance from a midwife and wanted to see a doctor or the midwife determined that a doctor's visit was necessary, there was no seamless connection directly to a doctor's visit. However, this connection could work if a clinic that offers online care works with a midwifery center.

In this context, we present the first case where online telemedicine for headaches reduced the need for patient travel by partnering with a midwifery center that offers online health guidance for patients who face difficulties visiting a hospital due to the postpartum period, childcare, and breastfeeding.

## Case presentation

A 29-year-old female consulted a midwife online. She and her husband were raising an 8-month-old infant in Sado City, an isolated island in Niigata Prefecture. The population of Sado City is 46,871 individuals. Although there is one general acute care hospital, the city is designated by the government as a "no-doctor area" or "quasi-no-doctor area (a district similar to a no-doctor area)" where healthcare measures for remote areas need to be implemented. The aging rate in 2020 will be about 42% (28.7% in Japan as a whole).

She developed acute lumbago and spent several days in bed due to difficulty moving her body. She consulted the midwife because of stress and anxiety caused by childcare and acute back pain, as well as newly developed headaches. The midwife provided online health guidance by listening closely and warmly. The midwife determined that the patient had acute back pain and tension-type headache attacks, so she instructed exercises for back pain and headaches online, but since the patient was too sore to practice them fully, she decided that a visit, including massage, would be better. After the midwife visits the patient, physical therapy for back pain and exercises for headaches [8] were lectured. Because there was little improvement in symptoms despite massages and exercises, the midwife decided that a doctor's diagnosis and treatment with painkillers were desirable for the headache and back pain, so she contacted a doctor based on the patient's request.

We, the physicians, provided online medical care across the sea from Nagaoka City, Niigata Prefecture. The patients presented the headache with a numeric rating scale of 4/10. The headache was not pulsatile but unilateral. The headache was not aggravated by physical activity, and nausea, phonophobia, or photophobia were confirmed. Similar headaches have been reported in the past, although they have been rare, and she forgot the details. The attack lasted for about six hours. We diagnosed acute lumbago and infrequent episodic tension-type headache (TTH) not associated with pericranial tenderness (the third edition of the International Classification of. Headache Disorders (ICHD-3) code 2.1.2) and prescribed acetaminophen 500 mg as an acute medication. The prescription was faxed to a pharmacy in Sado City (the original was mailed afterward), and the midwife picked up the medication and delivered it to the patient. Online medication guidance was performed. After taking the medication, the back pain and headache went into remission in 30 minutes. If there had been no improvement, the use of stronger non-steroidal anti-inflammatory drugs (NSAIDs) or intravenous acetaminophen would have been considered, but careful consideration was necessary because of the patient's lactation.

**Figure 1:**
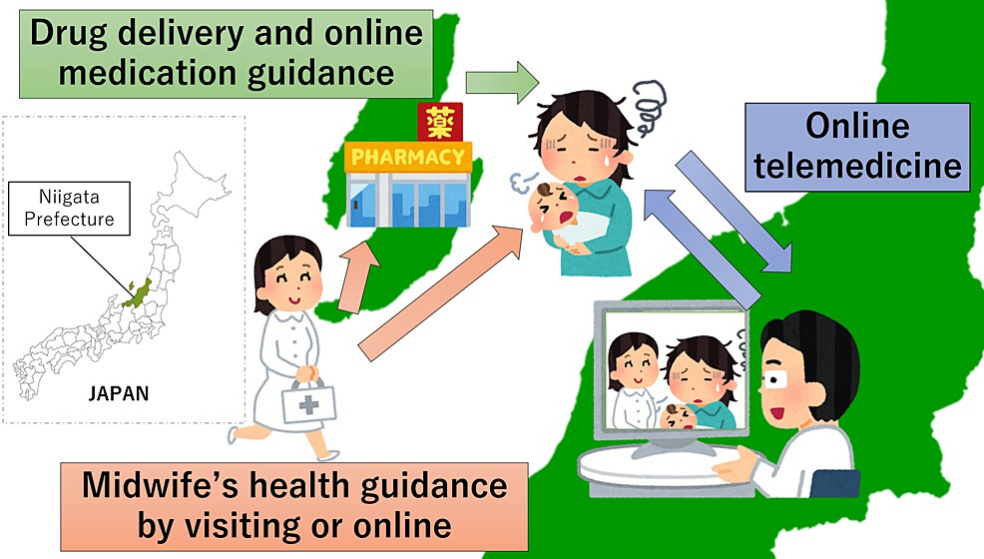
Illustration of online telemedicine in collaboration with a midwifery home The patient lived on a remote island. She consulted a midwife about her back pain and headache, and the midwife visited her. At the patient's request, online telemedicine across the sea was provided with a doctor on the mainland, and a pharmacy on the island arranged prescribed medications. The patient completed the medical treatment without leaving home. Image credit: Masahito Katsuki

Patients never had to leave their homes and could receive online consultations with midwives, home visits by midwives, and online telemedicine from physicians, all without leaving their homes. A geographical overview of this medical care is shown in Figure 1.

## Discussion

This is the first case where online telemedicine for headaches reduced the need for patient travel by partnering with a midwifery center that offers online health guidance for patients who face difficulties visiting a hospital due to the postpartum period, childcare, and breastfeeding. The novelty lies in the fact that midwives are at the core of this system, connecting doctors and patients, in addition to the usual online medical care provided by doctors and patients. If this collaboration between the midwife and doctors and online medical care were not available, the patient would have had to search throughout Japan for a clinic that offered online medical telemedicine or go to a clinic with back pain or headache.

Changes in online medical practice in Japan

In Japan, telemedicine has been discussed since 1997 but has not been widely used due to strict restrictions. 2018 saw the lifting of the ban on online medical care only for specific diseases like incurable diseases, but the utilization rate was only about 1/1 million. In April 2020, the prohibition of online medical care was lifted for some chronic diseases, only for follow-up visits, in order to help the medical system, which was under pressure due to the COVID-19 pandemic. Finally, in 2022, the ban on online medical care was lifted for all diseases, starting with initial consultations (Table 1).

**Table 1:** History of online telemedicine in Japan

Year	Event
1997	Officially approved with the following conditions: 'patients in remote islands and remote areas,' 'patients with certain chronic diseases,' and 'in principle, first face-to-face consultation.' Tele-radiography and tele-pathology were being performed quietly.
2015	Notified in 1997 that it is acceptable to provide telemedicine to patients with conditions other than those specified at the physician's discretion.
2018	Partial lifting of the ban on online medical care. Limited some diseases and only in the return visits. Patients should be able to access the hospital in 30 minutes.
2020	Expansion of some of the covered diseases and elimination of the 30-minute rule.
2022	Full lifting of the ban on online medical services for first-time patients and making it permanent.

The initial reason for the widespread use of online medical care was the need to avoid face-to-face treatment due to infectious diseases as much as possible and to avoid person-to-person contact due to hospital visits. In recent years, the need for online medical services has gradually shifted to other purposes. One is a specialized outpatient clinic. For example, there are no more than 1,000 headache specialists in Japan, and many patients cannot visit an outpatient headache-specialized clinic. For such patients, appropriate preventive treatment and patient guidance are provided through online medical services [3,4]. Online psychiatric care has also begun (https://fastdoctor.jp/mental/) because of the months-long waits for appointments to see a doctor. Psychiatrists throughout Japan with free time are task-sharing, and doctors are seeing patients throughout Japan. Prescription and monitoring through online telemedicine for obstructive sleep apnea with long-term continuous positive airway pressure [9] has also been practiced. Thus, some medical institutions are addressing unmet needs nationwide, sharing patients across the country, even if the patients do not have a specialty outpatient clinic nearby and cannot be seen.

The other is remote and rural medicine. Some remote islands have long offered online medical care only to return patients, but it has not been widely adopted, and many municipalities are having difficulty securing physicians. So far, physicians have made home visits in person, but a new form of medical care is beginning to emerge in which nurses visit alone, and physicians see patients from their clinics online. There was a significant negative correlation between the percentage of clinics offering telemedicine and population density (r = -0.31). The negative correlation between the provision of telemedicine in clinics and population density across Japan might reflect efforts to ensure that residents in less populated areas have access to medical services [10]. On the other hand, online medical care requires the use of a communication device, and some people, such as older people and those unfamiliar with device operation, are unable to receive online medical care [11]. Therefore, municipalities and hospitals began to prepare vehicles with nurses and simple laboratory equipment, which would visit patients' homes, and doctors would provide care online from the hospital. The nurses will help the patients to use the devices. There is an additional fee for this (Additional fee for remote medical assistance for nurses). The ban is also being lifted to allow patients to gather at community centers, schools, and other locations outside of their homes and hospitals to provide online medical care. In addition, a number of user-friendly products are being developed with video calling devices for online medical care. With the shortage of doctors and an aging population in Japan, online medical care is expected to be a breakthrough in equalizing medical care in various areas [12]. Further research is needed to determine its convenience and practical problems, and we should explore the future potential of online medical care in Japan, particularly in addressing healthcare disparities caused by physician shortages and an aging population, with a call for further research to optimize implementation and effectiveness.

Treatment needs for TTH

Headaches are a widespread public health issue, with primary types such as migraines, TTH, and trigeminal autonomic cephalalgias classified in the ICHD-3. In Japan, migraine prevalence ranges from 0.9% to 9.5%, TTH 15% to 20% of the population [13], and medication-overuse headache (MOH) 2.3% [14]. Approximately 22.4% to 29.2% of TTH sufferers report that TTH impairs their performance [15]. The Clinical Practice Guideline for Headache Disorders 2021 [16] outlines treatment strategies for TTH. Acute pharmacotherapy typically involves acetaminophen and non-steroidal anti-inflammatory drugs. For frequent episodic and chronic TTH, prophylactic treatment may include amitriptyline. Additionally, non-pharmacological treatments such as psychiatric care, psycho-behavioral therapy, physical therapy, and acupuncture should be considered for all TTH patients.

Our case was diagnosed as TTH after the online consultation and improved with acetaminophen. The treatment was appropriate because even TTH, not migraine, can interfere with life. The relative infant dose (RID) is a measure that estimates the degree of effect of drug exposure on infants via breast milk. The RID of acetaminophen is 2-4% [17], so acetaminophen is recognized as relatively safe for women who are breastfeeding. Our case suggests that acute headaches can be treated efficiently if the physician and patient are connected through the midwife’s coordination action and online telemedical system. Furthermore, integrated healthcare approaches, facilitated by midwives and telemedicine, can potentially improve treatment outcomes for headache disorders.

Midwife’s practice online

Traditionally, midwifery centers provide antenatal care, delivery services, and health guidance for various demographics. Midwives have a particular strength in providing various health guidance related to women, pregnancy, and delivery that obstetricians and nurses alone cannot provide. These activities have recently expanded into on-site and online platforms. Midwives provide some online practice, including individual antenatal education online [18,19] and developing mobile apps that could make telemedicine even more comfortable [20]. Such efforts online are widespread. However, they are intended only for health guidance and do not directly lead to medical treatment by physicians. In our case, the midwife took the lead in linking the patient to medical care. In this way, midwives and physicians can cooperate, and online medical care can be more convenient for patients.

Limitations and considerations

As a limitation, online telemedicine should be wary of secondary headaches because of the lack of adequate consultation and the little information that can be obtained. It should also be noted that online medical care is provided at the request of the patient, and midwives should not blindly arrange for consultations. Furthermore, the following points should also be considered: Technical issues and privacy/security concerns can hinder effective telemedicine services. Limited physical examination and dependence on technology can lead to missed diagnoses or inaccurate assessments. Patient engagement, adherence, and access to specialized care are significant considerations. Varying laws, regulations, and reimbursement models across regions and countries require careful navigation.

## Conclusions

This is the first case where online telemedicine for headaches has reduced the need for patient travel by collaborating with a midwifery center that provides online health guidance for patients who have difficulty visiting a hospital due to the postpartum period, childcare, and breastfeeding. The innovation lies in the central role of midwives, who connect doctors and patients alongside the online medical care provided by doctors. In the future, more non-physician medical professionals, such as nurses and midwives, may take the lead in connecting to online medical care. The online telemedicine service can be expanded to additional specialties and healthcare professionals to provide comprehensive care for patients. In parallel, a robust evaluation and feedback system should be developed to continuously assess the effectiveness and quality of the telemedicine service, identifying areas for improvement and ensuring patient satisfaction.
